# Opioid use in Latin America: Chronicle of a death foretold?

**DOI:** 10.7189/jogh.14.03040

**Published:** 2024-10-18

**Authors:** Paula J León, Fernando R Altermatt, Eduardo A Vega, María F Elgueta, Javiera Léniz

**Affiliations:** 1 División de Anestesiología, Escuela de Medicina, Pontificia Universidad Católica de Chile, Santiago, Chile; 2 Departamento de Salud Pública, Escuela de Medicina, Pontificia Universidad Católica de Chile, Santiago, Chile

## THE GLOBAL OPIOID CHALLENGE

Opioid drugs are valuable tools for pain management if administered and monitored correctly. However, when misused, opioids can cause significant harm to the patient and society [1]. In the last two decades, we have witnessed a dramatic expansion in opioid use and overuse in North America, with severe consequences for populations of the USA and Canada [2]. Moreover, the opioid crisis is now extending to other regions, including Africa, the Middle East, and Latin America [3]. This opinion piece explores global trends in opioid use, with a focus on Latin America. We analyse the current situation, drawing parallels and contrasts between different Latin American (LATAM) countries and the early stages of the crisis in North America 20 years ago.

Opioids number among the drugs recommended for pain management by the World Health Organization (WHO), and are included in the list of essential medicines for universal health care access. However, despite their broad efficacy as analgesics for surgical and cancer-related pain, opioids have a well-established addictive potential. Each type varies in potency and addictive potential, but even less potent opioids commonly used for pain management, such as tramadol, carry significant risks of dependency and overdose. These risks are even higher for the extremely potent fentanyl, a significant contributor to overdose deaths. People who consume opioid drugs are also more likely to use illicit drugs [1], such as the highly addictive and commonly abused drug heroin, which is often cheaper and more accessible.

Opioid use disorder (OUD) is a chronic disease associated with the misuse and/or abuse of opioid drugs [4,5] that can lead to dependence, withdrawal, adverse impacts on functionality, and overdose [6-8]. This condition often results from pain treatment appropriately prescribed by a physician, particularly in patients with chronic non-malignant pain. Over one-third of chronic pain patients treated with opioids develop problematic use patterns, and 8–12% become addicted [9]. Critically, many patients who initiate opioid treatments for pain management are unable to cease treatment and increase their doses progressively, inadvertently progressing toward addiction [10,11]. Thus, by the time an OUD becomes established, patients have usually experienced several weeks of problematic opioid use behaviour, during which time the problem could have been more readily addressed.

In 2020, almost two million people depended on or abused prescribed opioids [12]. Although it is difficult to assess the full extent to which medical use of prescribed opioids leads to non-medical opioid abuse [5,13-15], a rise in opioid prescriptions over the past several decades has fuelled an alarming increase in their problematic use [1]. From 1999 to 2021, nearly 280 000 people died in the USA from overdoses involving prescribed opioids, corresponding to nearly a 5-fold increase during this period. Moreover, prescribed opioids were involved in nearly 21% of all opioid overdose deaths in 2021, underscoring their role in this epidemic [16].

### Opioid use in Latin America

Average opioid consumption in Latin America and the Caribbean is moderate by international standards and significantly less than the alarming amounts consumed in other regions, such as North America or Africa [17]. However, observed usage trends in LATAM countries are concerning. Chile, Colombia, and Argentina have reported a 10-fold increase in opioid consumption over the past two decades. In parallel, the burden of OUD has increased steadily in this region within the last 30 years, with Chile and Uruguay exhibiting the highest rates of opioid use–associated disability [18].

Chile leads the region, consuming 1692 daily doses for statistical purposes (S-DDD) per million inhabitants per day, an amount still far below that reported by the USA (34 731 S-DDD per million inhabitants per day). Chile is followed by Colombia, Argentina, Uruguay, and Costa Rica, which consume 1434, 1050, 840, and 502 S-DDD per million inhabitants per day, respectively. In contrast, consumption is lower in LATAM countries with limited access to opioids, including Venezuela, Honduras, and Nicaragua, which consume 27, 40, and 64 S-DDD per million inhabitants per day, respectively [19]. Although not all LATAM countries report official statistics on OUD rates, available evidence suggests most patients suffering from this condition began by taking medically prescribed opioids without proper adherence to pain-management guidelines [12,20].

Despite witnessing the severe ramifications of opioid use in developed countries, there has been little effort to systematically address chronic pain management as a public health concern in LATAM countries. The International Narcotics Control Board (INCB) periodically publishes technical reports based on information provided by different governments [19], revealing that LATAM nations with limited access to opioids coexist with others in which these drugs are increasingly and liberally prescribed without adequate emphasis on rational use. For example, almost 5% of Uruguayans used prescription opioids in 2018 [21], and in 2020, opioid misuse was classified as an emergent drug problem in Uruguay by the Inter-American Commission for Drug Abuse Control and Uruguayan government.

Some LATAM countries have taken measures to address the problem. Brazil, for instance, recently implemented stringent regulations for opioid prescribing (e.g. restrictive prescription formalities, low-dose limits, prescriber limitations to authorised physicians and dentists) [22]. Although the country has a relatively moderate consumption of opioids compared to its neighbours, these regulations were spurred by a 2-fold increase in opioid consumption in the last 20 years concurrent with a transition from low-potency opioids like codeine to stronger ones, such as oxycodone [23]. Brazil also has the highest disability-adjusted life years due to OUD, together with the greatest availability and approval rates for methadone and naloxone programmes in the sub-continent [24]. Given these trends, more accurate indicators for opioid use, prescribing practice, and usage hazards in Brazil are required.

### The case of Chile

Chile represents one interesting study case. The INCB has reported an 8-fold increase in opioid consumption in Chile since 2000, an increase that mirrors those previously observed in high-income countries. Chile currently exhibits the highest per-capita opioid consumption in Latin America [19], far surpassing 200 S-DDD per million inhabitants per day, established by the WHO as the dose level sufficient to relieve pain in all areas [18]. Additionally, similar to high-income countries, there is another component to this issue in Chile that has not been properly addressed. That is, a 2018 report noted an OUD prevalence rate of 29% in a sample of patients with chronic non-cancer pain (CNCP), identifying tramadol, which is widely used in primary care for patients with knee and hip osteoarthritis and fibromyalgia, as the most frequently used opioid [25,26].

The reasons for the rising rates of opioid use and OUD in Chile are likely multifactorial. In 2016, an Advisory Panel of Experts in the management of chronic pain, ‘Change Pain Latin America,’ advocated for the safe and effective use of opioids for CNCP [27], and in 2017, the Latin-American guidelines for opioid use in CNCP were released [28]. These regional and national policies have expanded opioid analgesic availability in Chile and other LATAM countries without adequate training for health care professionals [29]. In particular, recently developed Chilean national guidelines for CNCP management recommend opioid use for conditions such as osteoarthritis, diabetic polyneuropathy, lumbar radiculopathy, complex regional pain syndrome, chronic postsurgical pain, and fibromyalgia. Consequently, opioids are now widely available in both primary and secondary health care settings [30]. Given the reported overall CNCP prevalence in Chile of around 34% [29,31], this policy has powerful implications for the number of people exposed to prescribed opioids and could at least partly explain the observed rise in opioid consumption numbers. Furthermore, according to the latest National Drug Survey performed in Chile in 2016–2018, the number of opioid analgesics consumed without a medical prescription quadrupled in the last decade. Opioids in Chile are most frequently accessed through a non-certified provider (30.0%) or family member/friend (24.8%), reflecting a lack of control over prescriptions, sales, access, and management of leftover doses, which has likely further contributed to the problem [31].

**Figure 1:**
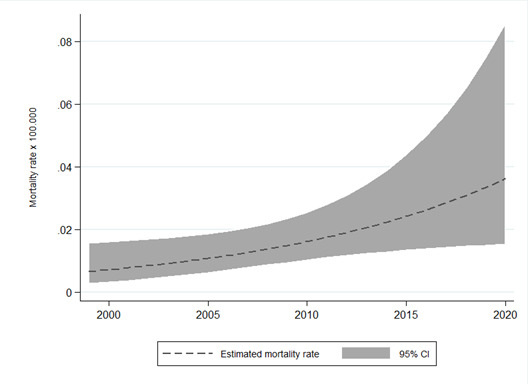
Mortality rates due to poisoning by narcotics and psychodysleptics drugs (ICD10 codes T40.0-T40.4; T40.6) for people aged 15 years and older between 1999 and 2020 in Chile. The graph was developed using data available online from Nationality records for the Chilean population obtained from the Department of National Health Statistics.

According to the United Nations Office on Drugs and Crime report, opioids are currently the third-most prevalent drug consumed in Chile, surpassing cocaine at the national level [32]. The same report notes an increase in the annual drug overdose mortality rate for both men and women between 2001 and 2015, placing Chile in the top half of countries included in the report. Moreover, although data from the Department of the National Health Statistics indicate that the narcotics mortality rate in Chile is low – estimated at 0.036 per 100 000 inhabitants in 2020 among those >15 years old – this rate is rising, increasing by 5-fold between 1999 and 2020 (**Figure 1**). Thus, despite absolute numbers that are far from catastrophic, the steady increase in opioid use, overdose rates, and mortality over the last 20 years is an alarm bell that should be taken seriously by health authorities.

### Facing the challenges of opioid consumption in Latin America

Given the epidemiological transition profiles facing some LATAM countries, it is likely at least part of the region will move toward an opioid consumption pattern similar to that observed in developed economies. Several LATAM countries have also repeated mistakes made in other regions, such as the widespread prescription of opioids in Chile in the 1990s for CNCP [26], contributing to the perception these drugs are harmless. Thus, there is clear and imminent risk of escalation in prescription opioid addiction and overdose in some regions of Latin America. Critically, however, LATAM countries can also learn important lessons from the North American crisis, wherein the liberal prescription of opioid analgesics was driven by an inflated perception of their benefits for pain management, coupled with an underestimation of their potential harm. Such fallacies may be avoided by ensuring opioid prescription guidelines in Latin America, South Africa, and India are free from bias and conflict of interest [33]. Unfortunately, this issue has already arisen, as the published WHO guidelines on increasing accessibility and availability of opioids in low- and middle-income countries were retracted after finding that experts involved in their development had conﬂicts of interest with pharmaceutical companies [34]. Similar concerns were also raised about usage guidelines in Latin America, South Africa, and India [3].

Current LATAM guidelines for opioid treatment in patients with CNCP mention ‘opiophobia’ as a barrier to effective pain management [28,35] and recommend tramadol for conditions such as fibromyalgia, chronic low back pain, and central neuropathic pain, among others, underestimating the risks of OUD development. However, the available evidence does not support use of tramadol as a first-line therapy for fibromyalgia [36] or chronic low back pain [37,38]. Furthermore, international evidence suggests tramadol increases risk of OUD, and several countries have established higher levels of control. Despite this shift, many LATAM countries are far from implementing such policies and continue to promote tramadol for prevalent chronic pain conditions [39–41].

The use of opioids for medical purposes requires a delicate balance between access and control. Improving evidence-based opioid access and availability for analgesic use must go along with training and regulations to avoid misuse and complications. To accomplish these objectives, countries should review and update their legislation and regulatory systems. Moreover, an adequate and well-resourced infrastructure is needed to ensure the provision and equitable distribution of medicines in diverse regions with variable barriers to access. Providing training for health care professionals can also reduce existing barriers to correct usage of opioids while preventing diversion for non-medical use [42].

## CONCLUSION AND RECOMMENDATIONS

‘Those who cannot remember the past are condemned to repeat it’ [43]. Although Latin America is not yet in an opioid crisis, it must heed the lessons of North America. Preventing uncontrolled expansion of opioid prescriptions in Latin America and, ultimately, a crisis similar to that experienced in other regions over the past two decades requires putting into practice a series of measures to promote their correct usage. Such guidelines should seek equity to ensure we do not abandon our patients by offering insufficient analgesia for their agony while establishing effective monitoring measures and non-punitive opioid distribution control. Most importantly, we must provide adequate education and training for health care professionals that is based on clear, robust, and bias-free evidence to maximise the well-being and safety of our patients.

## References

[1] Manchikanti L Cash KA Damron KS Manchukonda R Pampati V McManus CD Controlled substance abuse and illicit drug use in chronic pain patients: An evaluation of multiple variables. Pain Physician. 2006;9:215–25. 16886030

[2] Bohnert ASB Guy GP Losby JL Opioid Prescribing in the United States Before and After the Centers for Disease Control and Prevention’s 2016 Opioid Guideline. Ann Intern Med. 2018;169:367. 10.7326/M18-1243 30167651 PMC6176709

[3] Furlan AD Harvey AM Chadha R Warning from Canada: Latin America, South Africa and India may face an opioid epidemic in the coming years. J Glob Health. 2020;10:010324. 10.7189/jogh.10.010324 32257147 PMC7101494

[4] Smith SM Dart RC Katz NP Paillard F Adams EH Comer SD Classification and definition of misuse, abuse, and related events in clinical trials&colon; ACTTION systematic review and recommendations. Pain. 2013;154:2287–96. 10.1016/j.pain.2013.05.053 23792283 PMC5460151

[5] UNODC. World Drug Report 2023 (United Nations publication, 2023). 2023. Available: https://www.unodc.org/res/WDR-2023/WDR23_Exsum_fin_SP.pdf. Accessed: 13 August 2024*.*

[6] Atluri S Sudarshan G Manchikanti L Assessment of the trends in medical use and misuse of opioid analgesics from 2004 to 2011. Pain Physician. 2014;17:E119–28. 10.36076/ppj.2014/17/E119 24658483

[7] Manchikanti L Opioid Epidemic in the United States. Pain Physician. 2012;15:ES9–38. 10.36076/ppj.2012/15/ES9 22786464

[8] Birnbaum HG White AG Schiller M Waldman T Cleveland JM Roland CL Societal Costs of Prescription Opioid Abuse, Dependence, and Misuse in the United States. Pain Med. 2011;12:657–67. 10.1111/j.1526-4637.2011.01075.x 21392250

[9] Vowles KE McEntee ML Julnes PS Frohe T Ney JP van der Goes DN Rates of opioid misuse, abuse, and addiction in chronic pain. Pain. 2015;156:569–76. 10.1097/01.j.pain.0000460357.01998.f1 25785523

[10] Ballantyne JC LaForge SK Opioid dependence and addiction during opioid treatment of chronic pain. Pain. 2007;129:235–55. 10.1016/j.pain.2007.03.028 17482363

[11] Hser Y-I Evans E Grella C Ling W Anglin D Long-Term Course of Opioid Addiction. Harv Rev Psychiatry. 2015;23:76–89. 10.1097/HRP.0000000000000052 25747921

[12] Castaldelli-Maia JM Wang Y-P Brunoni AR Faro A Guimarães RA Lucchetti G Burden of disease due to amphetamines, cannabis, cocaine, and opioid use disorders in South America, 1990–2019: a systematic analysis of the Global Burden of Disease Study 2019. Lancet Psychiatry. 2023;10:85–97. 10.1016/S2215-0366(22)00339-X 36697127 PMC9870787

[13] Siegal HA Carlson RG Kenne DR Swora MG Probable relationship between opioid abuse and heroin use. Am Fam physician. 2003;67:942–945. 12643356

[14] Lankenau SE Teti M Silva K Bloom JJ Harocopos A Treese M Initiation into prescription opioid misuse amongst young injection drug users. Int J Drug Policy. 2012;23:37–44. 10.1016/j.drugpo.2011.05.014 21689917 PMC3196821

[15] Compton WM Jones CM Baldwin GT Relationship between Nonmedical Prescription-Opioid Use and Heroin Use. N Engl J Med. 2016;374:154–63. 10.1056/NEJMra1508490 26760086 PMC11784537

[16] CDC. Statistics NC for H. Wide-ranging online data for epidemiologic research (WONDER). Atlanta, GA: CDC, National Center for Health Statistics. Available: http://wonder.cdc.gov. Accessed: 13 August 2024*.*

[17] Cleary J Lima LD Eisenchlas J Radbruch L Torode J Cherny NI Formulary availability and regulatory barriers to accessibility of opioids for cancer pain in Latin America and the Caribbean: a report from the Global Opioid Policy Initiative (GOPI). Ann Oncol. 2013;24:xi41–50. 10.1093/annonc/mdt502 24285228

[18] Scholten WK Christensen A-E Olesen AE Drewes AM Quantifying the Adequacy of Opioid Analgesic Consumption Globally: An Updated Method and Early Findings. Am J Public Health. 2019;109:52–7. 10.2105/AJPH.2018.304753 30496006 PMC6301424

[19] International Narcotics Control Board. Narcotic Drugs. Estimated World Requirements for 2023. Statistics for 2021. Vienna: United Nations; 2022. Available: https://www.incb.org/documents/Narcotic-Drugs/Technical-Publications/2022/Narcotic_Drugs_Technical_Publication_2022.pdf. Accessed: 13 August 2024.

[20] Garcia-Orjuela MG Alarcon-Franco L Sanchez-Fernandez JC Agudelo Y Zuluaga AF Dependence to legally prescribed opioid analgesics in a university hospital in Medellin-Colombia: an observational study. BMC Pharmacol Toxicol. 2016;17:42. 10.1186/s40360-016-0087-4 27624605 PMC5022208

[21] INFORME FINAL - Compendio Informes Opioides Uruguay_28_02_20 (1).pdf. In: Problemas de drogas emergentes: Opioides en Uruguay. 2020. Available: https://www.gub.uy/junta-nacional-drogas/comunicacion/publicaciones/problemas-drogas-emergentes-opioides-uruguay. Accessed: 13 August 2024.

[22] Cleary J Lima LD Eisenchlas J Radbruch L Torode J Cherny NI Formulary availability and regulatory barriers to accessibility of opioids for cancer pain in Latin America and the Caribbean: a report from the Global Opioid Policy Initiative (GOPI). Ann Oncol. 2013;24:xi41–50. 10.1093/annonc/mdt502 24285228

[23] Maia LO Daldegan-Bueno D Fischer B Opioid use, regulation, and harms in Brazil: a comprehensive narrative overview of available data and indicators. Subst Abuse Treat Prev Policy. 2021;16:12. 10.1186/s13011-021-00348-z 33499891 PMC7836143

[24] Marín-Navarrete R Medina-Mora ME Pérez-López A Horigian VE Development and evaluation of addiction treatment programs in Latin America. Curr Opin Psychiatry. 2018;31:306–14. 10.1097/YCO.0000000000000434 29846265 PMC6860910

[25] Bilbeny N Miranda JP Eberhard ME Ahumada M Méndez L Orellana ME Survey of chronic pain in Chile – prevalence and treatment, impact on mood, daily activities and quality of life. Scand J Pain. 2018;18:449–56. 10.1515/sjpain-2018-0076 29886456

[26] Catalán VV González JCV Lara AC Riesgo de uso indebido de opioides prescritos en pacientes con dolor crónico no oncológico en un hospital de sistema mutual en Chile. Revista Soc Dolor. 2021;28:82–91.

[27] Furlan AD Reardon R Weppler C Group NOUG Opioids for chronic noncancer pain: a new Canadian practice guideline. CMAJ. 2010;182:923–30. 10.1503/cmaj.100187 20439443 PMC2882451

[28] Lara-Solares A Zamora CA Garca CA Garcia JBS Cook M del RB Sierra PB Latin-American guidelines for opioid use in chronic nononcologic pain. Pain Manag. 2017;7:207–15. 10.2217/pmt-2016-0065 28166710

[29] Joranson DE Improving Availability of Opioid Pain Medications: Testing the Principle of Balance in Latin America. J Palliat Med. 2004;7:105–14. 10.1089/109662104322737377 15000794

[30] Chile Ministry of Health. Technical guidance. Management of chronic non-cancer pain in people aged 15 and over, in primary care. Available: https://www.ached.cl/upfiles/userfiles/files/Orientacion-Tecnica.pdf. Accessed: 13 August 2024.

[31] Chile ON de DSN para la P y R del C de D y A (SENDA) M del I y SPG de. Décimo Cuarto Estudio Nacional de Drogas en Población General de Chile, 2020. 2021. Available: https://www.senda.gob.cl/wp-content/uploads/2022/01/Estudio-PG2020.pdf. Accessed: 13 August 2024.

[32] Chen Y Shiels MS Thomas D Freedman ND de González AB Premature Mortality From Drug Overdoses: A Comparative Analysis of 13 Organisation for Economic Co-operation and Development Member Countries With High-Quality Death Certificate Data, 2001 to 2015. Ann Intern Med. 2019;170:352. 10.7326/M18-2415 30422274 PMC8294457

[33] Humphreys K Avoiding globalisation of the prescription opioid epidemic. Lancet. 2017;390:437–9. 10.1016/S0140-6736(17)31918-9 28792397

[34] Dyer O WHO retracts opioid guidelines after accepting that industry had an influence. BMJ. 2020;368:m105. 10.1136/bmj.m105 31924609

[35] Rico MA Kraychete DC Iskandar AJ Colimon F Lara-Solares A Cantisani JAF Use of Opioids in Latin America: The Need of an Evidence-Based Change. Pain Med. 2016;17:704–16. 26700728 10.1111/pme.12905

[36] Macfarlane GJ Kronisch C Dean LE Atzeni F Häuser W Fluß E EULAR revised recommendations for the management of fibromyalgia. Ann Rheum Dis. 2017;76:318. 10.1136/annrheumdis-2016-209724 27377815

[37] de Campos TF Low back pain and sciatica in over 16s: assessment and management NICE Guideline [NG59]. J Physiother. 2017;63:120. 10.1016/j.jphys.2017.02.012 28325480

[38] Moscoso FM Sther PL Hidalgo BP Céspedes P Consideraciones para el uso de tramadol en dolor crónico no oncológico en APS. ARS Med Rev Cienc Médicas. 2024;49:55–63.

[39] McDiarmid T Mackler L Schneider DM Clinical inquiries. What is the addiction risk associated with tramadol? J Fam Pract. 2005;54:72–3. 15623411

[40] Rostam-Abadi Y Gholami J Amin-Esmaeili M Safarcherati A Mojtabai R Ghadirzadeh MR Tramadol use and public health consequences in Iran: a systematic review and meta-analysis. Addiction. 2020;115:2213–42. 10.1111/add.15059 32196801

[41] Salm-Reifferscheidt L Tramadol: Africa’s opioid crisis. Lancet. 2018;391:1982–3. 10.1016/S0140-6736(18)31073-0 29864013

[42] Berterame S Erthal J Thomas J Fellner S Vosse B Clare P Use of and barriers to access to opioid analgesics: a worldwide, regional, and national study. Lancet. 2016;387:1644–56. 10.1016/S0140-6736(16)00161-6 26852264

[43] Santayana G. The Life of Reason New York: Charles Scribner’s Sons;1905.

